# Acute myocardial infarction after batroxobin treatment of sudden sensorineural hearing loss: Case report

**DOI:** 10.1097/MD.0000000000042158

**Published:** 2025-04-11

**Authors:** Yinping Lu, Yingxin Liu, Weiliang Tang, Ping Liu

**Affiliations:** a Department of Otorhinolaryngology, Shaoxing People’s Hospital, Shaoxing, Zhejiang Province, China; b Department of Cardiology, Shaoxing People’s Hospital, Shaoxing, Zhejiang Province, China.

**Keywords:** acute myocardial infarction, batroxobin, fibrinogen, sudden sensorineural hearing loss, thrombosis

## Abstract

**Rationale:**

The drug batroxobin is utilized to enhance microcirculation by reducing fibrinogen levels, making it a treatment option for the acute stage of severe sudden sensorineural hearing loss (SSNHL). However, the efficacy of batroxobin defibrination remains controversial. It is crucial to also consider the potential thrombotic effects of batroxobin. This is the first report of acute myocardial infarction (AMI) in patients after treatment for SSNHL with batroxobin, aiming to enhance its clinical safety profile.

**Patient concerns:**

A 61-year-old male patient presented with severe SSNHL in the left ear. During hospitalization, the plasma fibrinogen level remained consistently low following batroxobin treatment and subsequently led to AMI.

**Diagnoses:**

SSNHL, AMI.

**Interventions:**

Percutaneous coronary intervention surgery confirmed thrombus blockage in the middle segment of the right coronary artery, and the symptoms were relieved after thrombus removal.

**Outcomes:**

The patient was discharged from the hospital on the 5th day after surgery without any recurrence of AMI up to now.

**Lessons:**

The pharmacology and toxicity of batroxobin are intricate, necessitating careful consideration of potential secondary thrombosis during treatment, particularly regarding fibrinogen levels.

## 1. Introduction

Sudden sensorineural hearing loss (SSNHL) is a prevalent emergency in the field of otorhinolaryngology, significantly impacting patients’ quality of life. Impaired cochlear microcirculation is considered the underlying etiological factor of severe SSNHL. Batroxobin, through its defibrination properties, can effectively reduce blood viscosity, enhance cochlear blood flow, and inhibit thrombus formation. Therefore, batroxobin holds promise as a potential therapeutic intervention for patients with SSNHL in China. However, clinical application of batroxobin primarily focuses on the risk of bleeding, while giving less consideration to the potential thrombolytic risks. This article presents a case study involving suspected coronary thrombosis after treatment for severe SSNHL using batroxobin and provides a comprehensive review of relevant literature.

## 2. Case report

The patient, a 61-year-old male, was admitted to the hospital on January 29, 2024. He presented with sudden left-sided sensorineural hearing loss accompanied by persistent tinnitus 1 week prior to admission. He denied any symptoms of otalgia or otorrhea and reported no episodes of vertigo. The patient had a medical history of hypertension for over a decade and was currently prescribed “Take Cinidipine every morning” for its management. Furthermore, he denied any past or present tobacco use, alcohol consumption, as well as any significant family medical history. Physical examination of the auricle, ear canal, and tympanic membrane revealed no discernible abnormalities. Otoacoustic emission testing indicated bilateral failure, while electroaudiometry demonstrated severe sensorineural deafness in the left ear with an average hearing threshold loss of 90 dB. The treatment plan consisted of a daily intravenous infusion of methylprednisolone at a dosage of 80 mg for 5 days, administration of puerarine at a dose of 0.4 g for 14 days, initial batroxobin infusion at a strength of 10BU followed by subsequent infusions every other day at a strength of 5BU. Prior to each use of batroxobin, fibrinogen levels were required to be checked and confirmed above the threshold value of 1 g/L; otherwise, administration was postponed until this criterion was met.

**Figure 1.**
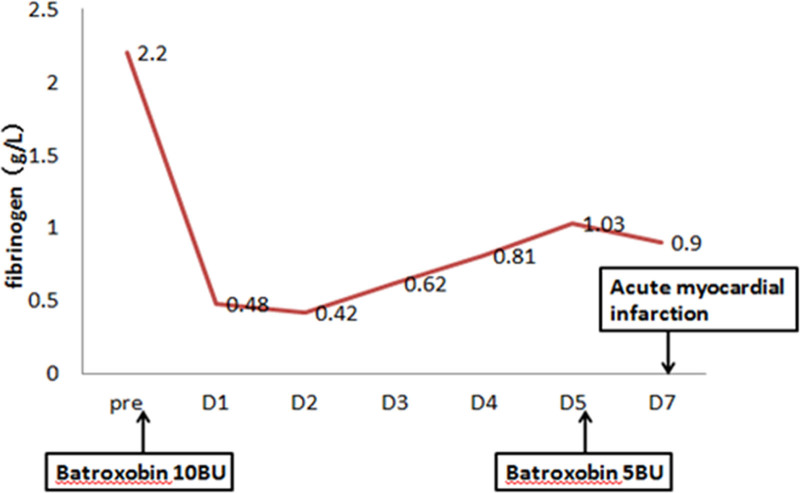
Throughout hospitalization, plasma fibrinogen monitoring revealed a consistently low level of fibrinogen (<1 g/L) following the initial administration of batroxobin 10BU, and subsequent occurrence of AMI after the second dose of batroxobin 5BU. AMI = acute myocardial infarction.

**Figure 2.**
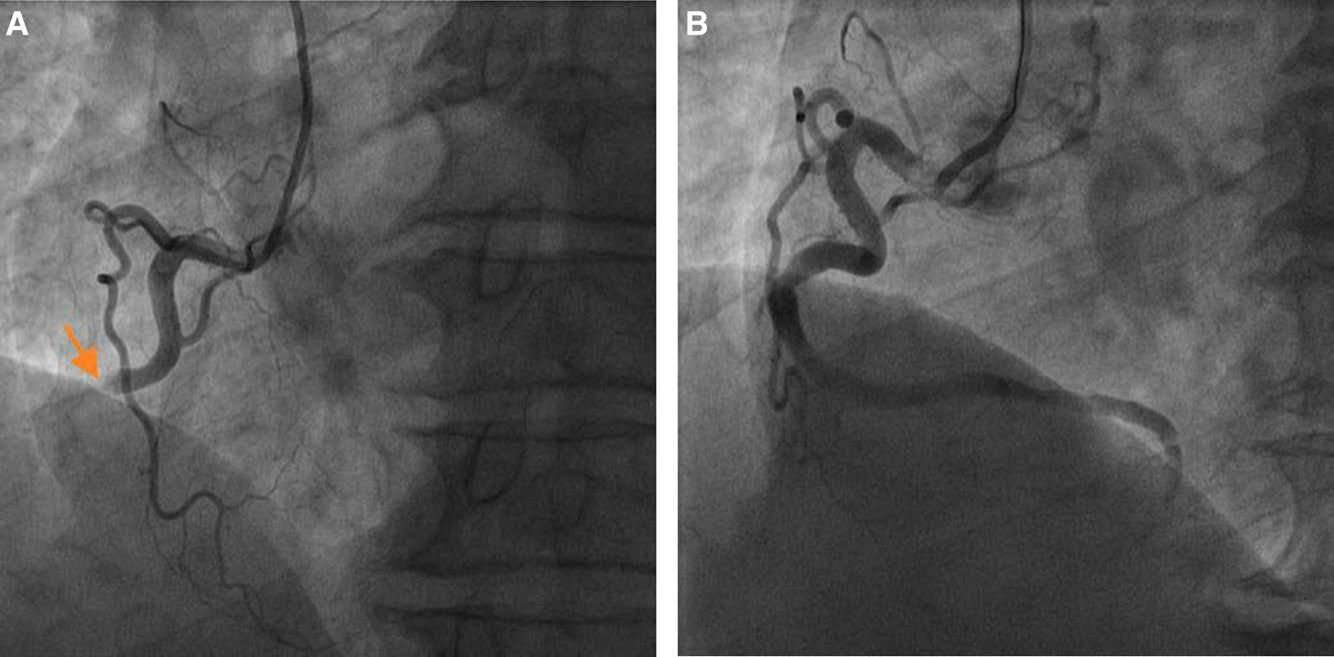
(A) Complete occlusion of the mid-segment of the right coronary stent is observed (indicated by the yellow arrow). (B) Blood vessel patency is restored after thrombus removal.

After the initial administration of batroxase 10BU, the patient exhibited a consistently low fibrinogen level, with the fibrinogen concentration only surpassing 1 g/L on the 5th day (Fig. 1). At approximately 8:20 am on the 7th day of hospitalization, the patient experienced a sudden loss of consciousness in the bathroom of the ward, accompanied by complaints of chest tightness, chest pain and fatigue. The immediate blood glucose level was measured at 2.1 mmol/L. Subsequently, oral glucose supplementation was administered to stabilize the patient’s condition while conducting an assessment including acute myocardial enzyme profile, troponin levels, d-dimer test and bedside electrocardiogram to evaluate for possible acute myocardial infarction (AMI). Following consultation with the patient’s family members who provided informed consent through signing documentation, percutaneous coronary intervention (PCI) was performed in the emergency department. The emergency procedure commenced at 10:50, revealing complete occlusion (100% stenosis) in the mid-segment of the right coronary artery. Subsequent dilation was performed using 8 atmospheric pressure balloons. After aspirating the thrombus and administering nitroglycerin for relief of vasospasm, luminal patency was successfully restored without requiring stent implantation (Fig. 2). The postoperative observation continued in the cardiology department, the patient was discharged from the hospital on the 5th day after surgery without any recurrence of AMI up to now.

## 3. Discussion

The pathogenesis of SSNHL is multifactorial, encompassing potential etiological factors such as viral infections, circulatory system disorders, autoimmune diseases, and metabolic disorders.^[1]^ In terms of its pathogenesis, severe SSNHL may potentially be associated with inner ear vascular embolism or thrombosis.^[2]^ The common cochlear artery serves as the principal blood supply to the cochlea, acting as a terminal artery without collateral circulation. Once embolized, this can lead to ischemia and hypoxia in the corresponding region, resulting in profound hearing impairment.^[3]^ Therefore, thrombolysis and defibrinogen have been proposed as potential treatments for severe SSNHL. However, the efficacy of these treatment strategies remains controversial. A study has reported no significant difference in short-term hearing improvement among patients with SSNHL who underwent defibrination therapy.^[4]^ The German guidelines for SSNHL, revised in 2014, do not recommend treatment options other than hormones,^[5]^ Similarly, the American Academy of Otolaryngology Head and Neck Surgery Foundation’s Clinical Practice Guidelines: Sudden Hearing Loss (2019) also do not endorse fibrinogen reduction as a treatment option for patients with SSNHL.^[6]^ However, several studies have demonstrated the efficacy of intravenous defibrinogenization drugs in treating patients with SSNHL.^[7,8]^ In contrast, the Chinese Guidelines for the Diagnosis and Treatment of Sudden Deafness (2015) recommend lowering fibrinogen levels as a treatment approach specifically for severe cases of SSNHL.^[9]^ This recommendation serves as the basis for administering batroxobin defibrination therapy to this patient.

Batroxobin is the generic name assigned by the World Health organization to the fibrin procoagulant proteinase found in Bothrops atrox snake venom. One subtype of batroxobin originates from Bothrops moojeni snake venom and has the function of degrading fibrinogen and anticoagulant thrombolysis.^[10]^ The other subtypes exhibit the function of promoting coagulation and halting blood flow.^[11,12]^ These 2 subtypes differ significantly in their physicochemical properties and clinical applications, which poses challenges for literature review and comprehension. To avoid confusion, batroxobin specifically refers to defibrase isolated from Bothrops moojeni snake venom in our country, representing a single-component serine protease. Fibrinogen exclusively serves as the substrate for batroxobin. In contrast to thrombin’s interaction with fibrinogen, batroxobin solely liberates fibrinopeptide A. The residual desA fibrin monomer undergoes conversion into a relatively loose fibrin polymer that is readily susceptible to degradation by the fibrinolytic system, thus achieving defibrination and reducing blood viscosity.^[10]^ Additionally, batroxobin activates endogenous t-PA-mediated thrombolytic mechanisms,^[13]^ also can counteract the anti-inflammatory effects of neutrophil extracellular traps through defibrinogenesis and promote ischemic tissue repair.^[14]^ Consequently, batroxobin finds application in the clinical treatment of ischemic cerebrovascular diseases and SSNHL.^[9,15,16]^ However, it is crucial to also consider the potential thrombotic effects of batroxobin. Despite the ease of decomposition of desA fibrin monomer polymer, there still exists a risk of its aggregation into thrombi at the site of vascular injury. Moreover, batroxobin exhibits significantly higher affinity for fibrin compared to thrombin and remains unaffected by antithromboprotein or heparin cofactor II inhibition, thereby promoting fiber adhesion.^[10]^ According to the literature, microvascular thrombosis has been reported in patients with Bothrops moojeni poisoning.^[17]^ When batroxobin is combined with other anticoagulant drugs for treating cerebral venous thrombosis, it can mitigate variations in coagulation index values caused by these drugs and prevent mutations in the coagulation system or bleeding events. This “remission effect” may be attributed to the procoagulant effect resulting from a high volume of fibrin degradation products produced after administration.^[18]^ Additionally, persistently low levels of fibrinogen increase the risk of thrombosis. It has been documented that hereditary hypofibrinogenemia can clinically manifest as either bleeding or thrombotic events within the same patient.^[19]^

**Figure 3.**
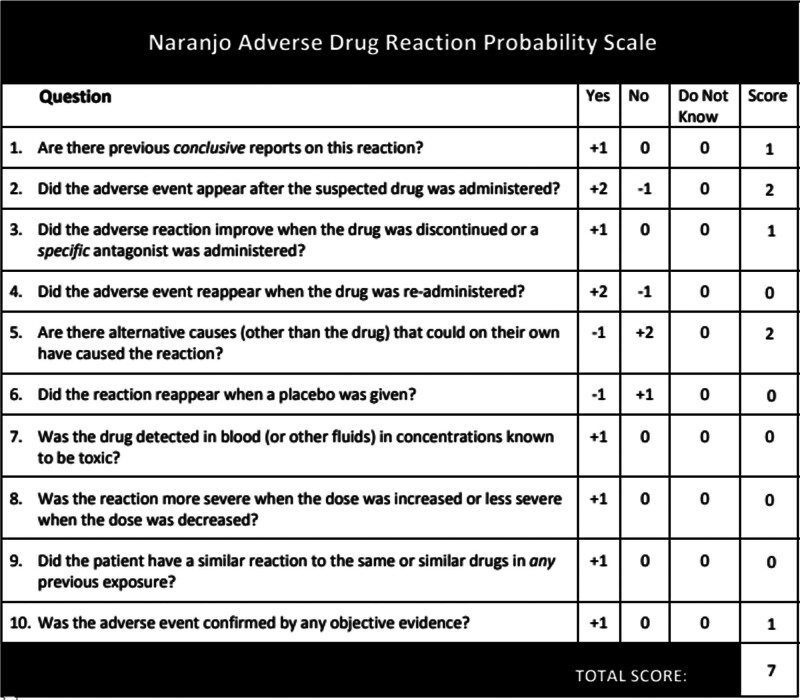
The total score of ≥ 9 indicates a positive causal relationship between the drug and adverse reactions. The total score of 5 to 8 suggests a likely relevance, supported by objective evidence or quantitative test results. The total score of 1 to 4 may indicate some relevance that cannot be fully confirmed or completely denied. An overall score of 0 or less is classified as suspicious, incidental, or largely irrelevant.

An analysis was conducted to investigate the etiology underlying AMI during the treatment course of this patient with SSNHL. The Naranjo Adverse Drug Reaction Assessment Scale yielded a score of 7 points (Fig. 3), suggesting a potential association between the occurrence of AMI and the administration of batroxobin. Firstly, the potential thrombolytic mechanism of batroxobin has been previously explained. SSNHL itself does not confer an increased risk of myocardial infarction.^[20]^ Through extensive retrospective cohort studies, Charlene Lin^[21]^ discovered that patients with SSNHL also exhibited a 1.39-fold elevated risk of subsequent myocardial infarction. Nevertheless, he attributed this association to shared etiology and risk factors between these 2 conditions, with more than 80% of cases occurring 1 year after the onset of SSNHL rather than during the acute stage. Secondly, the patient exhibited well-controlled hypertension in the absence of any other risk factors for coronary heart disease or a prior history of cerebrovascular disease or a family history. Lastly, following batroxobin administration in the patient’s case, abnormal plasma fibrinogen levels were observed, subsequently leading to the onset of AMI. PCI confirmed complete stenosis (100%) caused by thrombosis in the middle segment of the right coronary artery. The issue of coronary artery stenosis was resolved through removal of the thrombus during PCI intervention procedures, and subsequent coronary angiography revealed no apparent atherosclerotic lesions within blood vessels themselves.

## 4. Conclusion

Inner ear vascular embolism or thrombosis is considered a potential pathogenesis of severe SSNHL. However, the efficacy of batroxobin defibrination remains controversial. Blood anticoagulation and coagulation promotion involve intricate dynamics, while the pharmacology and toxicity of batroxobin are multifaceted. It is important to not only consider the risk of bleeding but also be cautious about the possibility of secondary thrombosis during thrombolytic therapy. Furthermore, special attention should be given to the persistent low level of fibrinogen after using batroxobin.

## Acknowledgments

I would like to express my gratitude to my supervisor, Prof Ping Liu, for his great support of my project. I would also like to thank the research team for their collaboration and help in gathering data for my research project.

## Author contributions

**Conceptualization:** Yinping Lu.

**Data curation:** Yingxin Liu, Weiliang Tang.

**Formal analysis:** Yinping Lu.

**Supervision:** Ping Liu.

**Visualization:** Yingxin Liu, Weiliang Tang.

**Writing – original draft:** Yinping Lu.
